# Editorial Note

**Published:** 1890-10

**Authors:** 


### EDITORIAL NOTE.

THE twenty-seventh annual meeting of the United States Veterinary Medical Association, held in Chicago on September 16th and 17th, proved so successful in bringing out a large number of interesting papers, valuable committee reports and active discussions, that the material furnished by it for publication proves too voluminous to be put in type hastily, and in time for the regular publication of the Journal; we, however, present our readers in this number with the papers read before the Association, the list of officers elected, committees appointed, new members elected and members dropped from the roll of the Association.

In our next number we will give in full the proceedings of the Association, including reports of committees and the discussion of papers and reports.

